# Targeting stromal components in pancreatic ductal adenocarcinoma: a review

**DOI:** 10.3332/ecancer.2025.1996

**Published:** 2025-09-24

**Authors:** Hani Shihadeh, Ahmad Yousef, Ahmad Al-Leimon, Hussein Abu-Rumman, Laith Kreshan

**Affiliations:** School of Medicine, University of Jordan, Amman 11118, Jordan

**Keywords:** pancreatic ductal adenocarcinoma, tumour stroma, cancer-associated fibroblasts, stroma-targeted therapy, tumour microenvironment

## Abstract

Pancreatic ductal adenocarcinoma (PDAC) remains a leading cause of cancer-related mortality, largely due to a lack of highly safe and effective therapeutic options. A large proportion of the tumour's mass consists of a dense fibrous stroma, which could provide valuable therapeutic targets. This review elucidates the various possible stromal targets in PDAC, methods of targeting them and the outcomes of this targeting in *in-vitro* studies, studies on murine models of PDAC and clinical studies. While targeting some stromal components in PDAC yielded disappointing results in clinical studies, others have shown promise in multiple settings. More research efforts should be directed towards identifying additional stromal targets and evaluating their therapeutic potential. In addition, comprehensive clinical studies are essential to evaluate the safety and effectiveness of agents targeting stromal components of PDA of agents targeting stromal components of PDAC, both as monotherapies and in combination with standard surgical and pharmacological treatments for PDAC to improve patient’s outcomes.

## Introduction

The prevalence of pancreatic cancer has increased twofold during the last 25 years. Researchers have attributed this increase to many factors, including rising life expectancies globally and rising rates of smoking, obesity, diabetes and alcohol consumption, all of which are known risk factors for developing pancreatic ductal adenocarcinoma (PDAC) [1]. Pancreatic cancer is known for its abysmal prognosis, exhibiting a 5-year survival rate below 15% [2]. Thus, it is a major cause of cancer-related mortality and a considerable public health problem. Despite significant progress in the treatment of several prevalent cancers, such as lung cancer, breast cancer and hematologic malignancies, there have been no substantial breakthroughs in the management of pancreatic cancer in recent years, resulting in persistently high mortality rates.

Surgery, radiation therapy and chemotherapy are the major therapeutic modalities available to prolong survival and alleviate symptoms in patients with PDAC. The only potentially curative therapy for pancreatic cancer is surgical resection with or without adjuvant chemotherapy [3]. However, approximately 80% of patients with PDAC seek medical attention only after the disease is either locally advanced or metastatic stage, rendering them ineligible for curative-aim surgery. In those patients, palliative chemotherapy is the mainstay of treatment [4]. Gemcitabine monotherapy, gemcitabine in combination with nanoparticle albumin-bound paclitaxel (Nab-paclitaxel) or the FOLFIRINOX regimen (a four-drug combination of fluorouracil, leucovorin, oxaliplatin and irinotecan) are utilised as chemotherapy options for PDAC. However, these regimens have limited efficacy and considerable toxicity. Therefore, there is an urgent necessity to devise innovative therapies for pancreatic malignancies that offer improved efficacy and reduced toxicity [5].

PDAC is known for its densely fibrous stroma, which constitutes a significant portion of the tumour mass, potentially reaching up to 90% [6]. This stroma is composed of cancer-associated fibroblasts (CAFs), which are thought to be derived from pancreatic stellate cells (PSCs) and the extracellular matrix (ECM). The ECM is composed of collagen fibers, other fibers and a ground substance rich in proteoglycans and glycosaminoglycans (GAGs). Hyaluronic acid (HA) is the most abundant GAG in the pancreatic cancer stroma [7, 8]. The dense fibrous nature of stroma is likely responsible, at least partially, for many characteristics of PDAC, including clinical aggressiveness and resistance to chemotherapy, while also presenting as a potential therapeutic target. Efforts by researchers to completely ablate the tumour stroma in PDAC murine models led to more aggressive tumour behaviour and diminished survival rates in the mice [9, 10], suggesting that the tumour’s stroma might actually play a tumour-restraining role. Current efforts focus on developing novel stroma-targeting therapies that would reprogram the stroma in an advantageous manner and avoid the extremes of depletion and abundance. In this review, we aim to summarise the latest insights on targeting the stromal components of PDAC, including *in vitro* studies, studies on murine models, preclinical and clinical studies, focusing on their most relevant advancements and limitations.

## Targeting HA

### Background

HA is an essential glycosaminoglycan involved in maintaining connective tissue functionality [11]. In PDAC, HA is synthesised by CAFs and is 12 times greater in quantity than in a healthy human pancreas [12]. HA serves many functions supporting PDAC progression, including acting as a reservoir for growth factors and cytokines [13, 14]. In addition, binding of HA to its receptors, CD44 and RHAMM on tumour cells, activates many oncogenic pathways that promote PDAC cell proliferation, invasion and metastasis [15–17]. Being highly hydrophilic, HA attracts water and which increases the interstitial fluid pressure (IFP) within the tumour microenvironment [18]; the high IFP compresses tumour vasculature and results in decreased local bioavailability of systemically administered drugs in the tumour microenvironment [19, 20]. Clinical data also support the role of HA in tumour progression. PDAC patients with lower levels of HA in their tumours had a significantly increased overall survival (24.3 median survival time versus 9.3 months in patients with high HA levels, *p* < 0.05) [21]. Table 1 summarises the results of targeting HA in PDAC.

### Effects of targeting the HA in preclinical studies

Researchers investigated the therapeutic potential of enzymatic remodeling of the tumour stroma by HA depletion using recombinant human hyaluronidase (rHuPH20); This enzyme degrades HA-dependent tumour ECM both *in vitro* and *in vivo*. Because rHuPH20 exhibits a short plasma half-life of less than 3 minutes following intravenous administration, a pegylated version (PEGPH20), with an improved half-life (10.3 hours), was developed and evaluated [14]. Depleting HA via hyaluronidase decreases the pressure within the tumour microenvironment and enhances intratumoural bioavailability of chemotherapeutic drugs [14, 18, 19, 22].

The therapeutic potential of combining the PEGPH20 with either gemcitabine or the FOLFIRINOX regimen was evaluated in many preclinical and clinical trials. When compared to gemcitabine alone, treatment with PEGPH20/gemcitabine combination significantly enhanced tumour suppression and prolonged survival in murine models of PDAC (median survival 15 days in PEGPH20/gemcitabine group versus 9 days in gemcitabine alone group,* p* = 0.0002) [19].

Researchers also examined whether hyaluronidase enhances could enhance the antitumour effect of immune checkpoint inhibitors. They hypothesised that hyaluronidase would enhance the intratumoural bioavailability of systemically administered immune checkpoint inhibitors or eliminate HA's possible immunosuppressive effects. Blair *et al* [23] found that adding PEGPH20 to an antibody against the immune checkpoint protein programmed death ligand-1 (PD-1) resulted in increased survival of murine models of PDAC and enhanced effector T cell infiltration of the tumour.

The rationale of using microbial vectors to deliver hyaluronidase into tumours is to provide preferential degradation of HA in the tumour without affecting that of normal tissues, thus achieving better efficacy and less toxicity. Ebelt *et al* [24] developed a novel, promising strategy for administering hyaluronidase using a live attenuated strain of the bacterium *Salmonella typhimurium*; this bacterium expresses a functional bacterial hyaluronidase; they colonise murine models of PDAC tumours following intravenous administration and induce tumour-specific HA depletion. Potentiated the cytotoxic effect of gemcitabine in these models [24].

VCN-01 is a genetically modified oncolytic adenovirus designed to proliferate only within tumour cells, sparing normal cells and express human hyaluronidase. In combination with gemcitabine, VCN-01 induced responses when administered intratumourally using endoscopic ultrasound in few patients [25].

Kudo *et al* [26] identified a novel phenotype of PDAC in both patients' samples and cell lines, called the HA activated-metabolism phenotype. This phenotype is characterised by high expression of genes involved in HA metabolism. They observed that patients with this phenotype of PDAC had a shorter overall survival. They also observed that 4-methylumbelliferone (4-MU), a synthetic inhibitor of hyaluronan synthase, inhibited the migration of PDAC cells *in vitro* [26].

4-MU also enhanced the anticancer effects of γδ T-cell immunotherapy in murine PDAC models by reducing the amount of ECM and increasing the infiltration of tumour-infiltrating lymphocytes [27]. Further research is needed to determine the efficacy and safety of 4-MU in human subjects.

### Effects of targeting the HA in clinical studies

A phase Ib single-arm trial conducted in 2016 evaluated the safety and tolerability of PEGPH20 with gemcitabine. The combination demonstrated tolerability and potential therapeutic advantages in patients with advanced PDAC, particularly in those with tumours with high HA levels [28]. A HALO 202 trial, a randomised Phase II trial assessed the efficacy and tolerability of PEGPH20 in combined with Nab-Paclitaxel/Gemcitabine versus Nab-Paclitaxel/Gemcitabine alone in treatment-naïve PDAC patients; it revealed that the addition of PEGPH20 significantly improved the progression-free survival (PFS) (hazard ratio, 0.73; *p* = 0.049), especially in patients with high HA levels (HR, 0.51; *p* = 0.048). However, it failed to improve overall survival [29]. These trials showed that thromboembolic diseases, including venous thrombosis and cerebrovascular accidents, were the most common severe adverse effects in patients receiving PEGPH20. However, it is unclear whether thromboembolic disease occurred at significantly higher rates in patients treated with PEGPH20 compared to patients receiving standard therapy, especially since PDAC is a malignancy that is frequently linked to thromboembolic disease [28–30]. A randomised double-arm phase III trial compared PEGPH20 combined with nab-paclitaxel/gemcitabine to nab-paclitaxel/gemcitabine alone; it revealed no significant increase in the overall survival or PFS of PDAC patients with added PEGPH20. Addition of PEGPH20 was also associated with significant adverse effects [31]. A randomised double-arm phase Ib/II clinical trial examined PEGPH20 combined with FOLFIRINOX versus FOLFIRINOX alone in patients with metastatic PDAC who had not received any prior therapy. Unexpectedly, adding PEGPH20 to the FOLFIRINOX regimen decreased overall and PFS (HR, 2.07; *p* < 0.01) [32].

The MORPHEUS-PDAC randomised trial evaluated the combination of Atezolizumab, an anti-PD-L1 monoclonal antibody, with PEGPH20 compared to standard chemotherapy in patients with previously treated metastatic PDAC. It revealed that patients receiving Atezolizumab plus PEGPH20 had similar overall survival and PFS to those receiving standard chemotherapy. However, in Patients with high HA content, the combination therapy was associated with significantly longer overall survival compared to treatment with standard chemotherapy (HR = 0.41, 95% CI (0.20–0.84)) [33], suggesting a potential benefit in this biomarker-defined subgroup.

## Targeting sonic hedgehog (SHh) pathway

### Background

The SHh pathway is a biochemical pathway normally involved in embryonic organogenesis. It involves the Shh ligand, which is released from cells to interact with its cell surface receptor on neighboring cells known as the patched protein (PTCH); this binding results in the disinhibition of smoothened protein (SMO) in target cells, SMO then activates downstream pathways that ultimately result in activation of Gli1, Gli2 and Gli3 transcription factors, which in turn affect the target cell’s transcriptional activity. Activation of the SHh pathway is common in many cancers, including PDAC [10, 34]. Specifically, a paracrine loop in which SHh ligand is secreted from tumour epithelial cells to activate the SHh pathway in PSCs was identified in both murine models and patients’ samples of PDAC [35]. SHh pathway activates PSCs to synthesise more tumour stroma [36, 37]. The clinical literature supports the contribution of SHh in PDAC, as high expression of SHh ligand and Gli1 are predictive of shorter patient survival [38]. Table 2 summarises the results of targeting SHh pathway in PDAC.

### Effects of targeting the SHh pathway in preclinical studies

Rhim *et al* [10] produced a murine PDAC model in which the gene coding for the Shh ligand was knocked out. Mice lacking the SHh gene developed tumours with a smaller stroma and had fewer activated PSCs. However, compared to mice with wild-type SHh PDAC, mice with knocked-out PDAC had shorter overall survival, suggesting that the Shh pathway, at least in these models, acts to impede tumour growth rather than to support it [10].

Cyclopamine, a naturally occurring steroidal alkaloid that binds and thus inhibits the SHh signaling pathway and blocks the SMO signaling transducer; when tested in a xenograft murine PDAC model, cyclopamine reduced the size of the tumour stroma and potentiated the antitumour effect of nab-paclitaxel, possibly due to enhanced drug delivery [39].

When combined with gemcitabine, Saridegib induced a temporary elevation in intratumoural vascular density and intra-tumoural gemcitabine bioavailability, leading to transient stabilisation of disease in murine PDAC models [40].

Itraconazole is a common antifungal drug; it inhibits ergosterol synthesis in fungi by targeting the cytochrome P450 enzyme 14-α-demethylase. Recently, researchers found that itraconazole inhibits the SHh pathway by inhibiting SMO by a distinct mechanism from that of cyclopamine and its derivatives [41, 42]. The antitumour effect of itraconazole was demonstrated *in vitro* [43, 44]. When combined with paclitaxel in a poly (ethylene glycol)-b-poly (D, L-lactide) micelle delivery system, itraconazole successfully inhibited the growth of a PDAC cell line *in vitro* and in murine models. This inhibition was partially due to the inhibition of the SHh pathway [45]. Itraconazole also inhibited migration and epithelial-to-mesenchymal transition of PDAC cell lines *in vitro* by inhibiting the SHh pathway and other mechanisms [46].

There has been a growing interest in inhibiting the SHh pathway downstream of SMO, largely because some tumours, including PDAC, have shown activation of the Gli transcription factor independent of SMO [47, 48]. Oxy186, a novel chemical compound with favourable pharmacokinetic properties, directly inhibits Gli transcription factors. It inhibits the proliferation of PDAC cell lines *in vitro* [49].

Taxanes are a family of cytotoxic drugs whose main mechanism of action is hyperstabilisation of polymerised microtubules, leading to inhibition of mitosis. Of these, nab-paclitaxel is an approved treatment for PDAC in combination with gemcitabine. Mohelnikova-Duchonova *et al* [34] identified a novel mechanism of action of taxanes, as they observed that a new generation taxane, SB-T-1216, strongly inhibited the Shh pathway *in vitro* and murine models of PDAC.

Aberrant epigenetic modifications are now widely recognised as a hallmark of cancer [50]. PDAC development heavily relies on epigenetic modifications, particularly DNA methylation, which suppresses gene expression and transcription. This is evident by the fact that DNA methyltransferase 1 (DNMT1), a major enzyme involved in DNA methylation, is commonly overexpressed in human PDAC [51]. In addition, many tumour suppressor genes are aberrantly hypermethylated, and thus suppressed in PDAC [52]. On the other hand, many proto-oncogenes are aberrantly hypomethylated, and thus activated [53].

DNMT1 inhibition could suppress Hedgehog pathway activation and decrease tumour growth. n-Butylidenephthalide, an inhibitor of DNMT1, inhibits proliferation and induces apoptosis of the PDAC cell line *in vitro*. This inhibition was mediated by hypomethylation and induction of the PTCHD4 gene, which codes for the PTCH protein. Increased expression of PTCH proteins leads to more effective sequestering of SMO and, thus, inhibition of the SHh pathway [54].

TET1, also known as ten-eleven translocation cytosine dioxygenase, is a major enzyme involved in the demethylation of DNA. TET1 promotes the expression of the Gli1 transcription factor in PDAC, thus activating the SHh. Inhibition of SHh by vismodegib reversed the chemoresistance mediated by TET1 in PDAC cell lines, suggesting that the SHh pathway significantly contributes to the chemoresistance of PDAC [55].

### Effects of targeting the SHh pathway in clinical studies

In a pilot single-arm clinical trial, a combination of vismodegib and gemcitabine was tested in patients with metastatic PDAC; the combination stabilised the disease in a few patients. However, nearly half of the patients experienced grade III adverse effects. The most common of which were anemia and elevated liver enzymes [56]. Another randomised double-arm Phase Ib/II trial compared gemcitabine/vismodegib combination to gemcitabine alone, it revealed that the combination had no superior response rates, PFS or overall survival [57].

Sonidegib was also investigated in combination with chemotherapy. A single-arm Phase I/II study for patients with metastatic PDAC setting sonidegib with gemcitabine/nab-paclitaxel after FOLFIRINOX treatment. The combination stabilised the disease in 58% patients and achieved a partial response in 13% patients. However, the treatment was associated with severe toxicities in some patients [58]. Sonidegib was evaluated with the FOLFIRINOX regimen in a single-arm phase 1b trial; this trial enlisted PDAC patients with locally advanced or metastatic disease who had not received previous chemotherapy. The combination stabilised 46% of patients and induced partial responses in 31% of patients [59].

On this basis, a phase Ib/II trial commenced to evaluate the safety and efficacy of Saridegib/gemcitabine combination with gemcitabine in metastatic PDAC. It showed that the combination was tolerable without unexpected toxicity, with preliminary evidence of clinical activity [60]. Similar findings were reported by Jimeno *et al* [61] in another phase I study. In a phase Ib study, Saridegib was evaluated in combination with FOLFIRINOX in patients with advanced pancreatic cancer. The treatment induced objective responses and decreased CA 19–9 levels, with most patients experiencing grades I–II adverse effects [62].

## Targeting secreted protein acidic and rich in cysteine (SPARC)

### Background

SPARC, also called osteonectin or basement membrane protein 40 (BM-40), is an ECM glycoprotein; it serves many physiological functions in the ECM, including bone mineralisation [63], sequestration of growth factors, cellular differentiation, migration and tissue repair [64]. SPARC

is expressed in the primary and metastatic PDAC lesions of PDAC several fold more than normal pancreatic tissue [65–67], and expression was predominantly localised in the tumour stroma. Patients with PDAC exhibiting high SPARC expression had a shorter overall survival than those with low SPARC expression (11.5 versus 25.3 months; *p* = 0.02) [65]. Table 3 summarises the results of targeting SPARC along with other stromal components in PDAC.

### Effects of targeting SPARC in preclinical studies

Albumin, the most abundant human plasma protein, binds to SPARC with high affinity. It is plausible that coupling albumin with chemotherapeutic drugs would enhance the intratumoural bioavailability of these drugs in tumours expressing high SPRAC [68]. In PDAC, the high expression of PDAC might explain the clinical success of nab-paclitaxel, a specialised paclitaxel formulation conjugated with albumin in nanoparticles. Nab-paclitaxel succeeded in preclinical and clinical studies [69–72].

### Effects of targeting the SPARC in clinical studies

Based on the results from the MPACT trial, which revealed that nab-paclitaxel/gemcitabine combination improved overall survival in patients with PDAC compared to gemcitabine alone (HR = 0.72, *p* < 0.001), the combination was FDA-approved [73]. Despite these findings, Hidalgo *et al* [74] reported that the expression of SPARC in patients’ sample and murine models of PDAC was not predictive of overall survival or efficacy of nab-paclitaxel [74].

## Targeting angiotensin II (AT II)

### Background

AT II is an octapeptide produced in the blood to serve many physiological functions, including blood pressure regulation, plasma potassium concentration and cardiac contractility. In addition to these widely recognised functions. AT II is involved in many processes in PDAC, it directly stimulates PSCs proliferation *in vitro* by activating the epidermal growth factor/extracellular signal-regulated kinase (ERK) pathway [75, 76]. Moreover, AT II receptor signaling inhibits the growth of pancreatic carcinoma cell both *in vitro* and in murine models of PDAC [77]. In addition, AT II stimulates the secretion of transforming growth factor-β (TGF-β), which activates PSCs to synthesize stromal components [78]. AT II can be easily targeted by the antihypertensive medications Angiotensin-converting enzyme inhibitors (ACEis) and AT II receptor blockers (ARBs).

### Effects of targeting the AT II in preclinical studies

Several preclinical studies on PDAC models showed that losartan, a selective and competitive ARB, reduced stromal collagen and HA production when combined with gemcitabine. In addition, losartan inhibited angiogenesis by reducing the expression of vascular endothelial growth factor (VEGF) and enhanced intratumoural gemcitabine bioavailability [76, 79–83].

### Effects of targeting the AT II in clinical studies

A large population study suggested that intake of ARBs and ACEis in patients with PDAC is associated with improved survival (HR = 0.80; 95% CI: 0.72, 0.89). However, the results may be due to the confounding beneficial cardiovascular effects of these medications [84]. A retrospective study found contradictory results; indicating no difference in survival and response rates between individuals administered losartan for any indication at the time of PDAC diagnosis and those who were not treated with it [85].

A single-arm clinical trial examined losartan, FOLFIRINOX and radiotherapy use in patients with unresectable, locally advanced, nonmetastatic PDAC; the regimen successfully downstaged the tumour so that it became resectable in 69% of patients. However, it remains to be determined whether losartan contributes to this benefit due to the nonrandomised design of the trial [86]. Another single-arm phase II trial evaluated gemcitabine in combination with candesartan for patients with treatment-naïve unresectable PDAC, the combination was not effective [87].

## Targeting matrix metalloproteinases (MMPs)

### Background

MMPs, a family of zinc-dependent proteolytic enzymes that degrade ECM proteins, facilitating tumour invasion and metastasis [88]. MMPs are normally kept in check by inhibitor proteins called Tissue inhibitors of matrix metalloproteinases (TIMPs). In PDAC, analysis of patients’ samples revealed that PSCs are a major source of MMPs and they are secreted in abundance at the invasive front of tumours. Secretion of MMPs by PSCs is stimulated by various paracrine signals from nearby carcinoma cells, including SHh ligand and cytokines such as IL-1 and TGF-β [89–92]. Eight types of MMPs were differentially expressed in PDAC, including MMP1, MMP2, MMP7, MMP9, MMP11, MMP12, MMP14 and MMP28. Patients with higher MMP1, MMP2, MMP7 and MMP9 expression had a worse prognosis [93]. PDAC also showed a loss of expression of TIMP2, which contributes to dysregulation of multiple MMPs in PDAC [94].

### Effects of targeting the MMPs in clinical studies

In a Phase I clinical evaluation, marimastat, a soluble, broad-spectrum MMP inhibitor, increased overall survival comparable to gemcitabine [95]. However, the combination therapy of gemcitabine and marimastat had no significant improvement in overall survival compared to gemcitabine alone [96].

A phase III trial compared the efficacy and safety of Tanomastat (BAY12-9566), an oral MMP inhibitor with selectivity toward MMP-2, MMP-3 and MMP-9, to gemcitabine monotherapy in 277 patients with advanced pancreatic cancer. Tanomastat was less effective and more toxic than gemcitabine [97].

## Pirfenidone

### Background

Pirfenidone is an FDA-approved antifibrotic drug used for the management of idiopathic pulmonary fibrosis. It inhibits fibrosis by multiple mechanisms, including inhibiting TGF-β-stimulated collagen synthesis [98], fibroblast activation and proliferation [99], inflammation and angiogenesis [100].

### Effects of pirfenidone in preclinical studies

The ability of pirfenidone to inhibit the proliferation of PDAC cells was confirmed by many *in vitro* and murine model studies. Multiple mechanisms were observed, including induction of cell cycle arrest [101], inhibition of TGM2/ NF-κB/PDGFB pathway [102], inhibition of CAFs proliferation [103] and enhancing drug delivery [103, 104]. Pirfenidone was never evaluated in a clinical study for the treatment of PDAC.

## Targeting connective tissue growth factor (CTGF)

### Background

CTGF, also called Cellular Communication Network 2 (CCN2), is a cysteine-rich matricellular protein that is involved in many cellular functions, including cellular adhesion, migration, proliferation and differentiation [105]. Studies on tumour samples from patients revealed that CTGF is overexpressed in PDAC compared to the normal pancreas [106], and it contributes to autocrine and paracrine pathways which promote cellular growth, invasion, metastasis and angiogenesis *in vitro* and murine models of PDAC [107].

### Effects of targeting the CTGF in preclinical studies

The main therapeutic approach designed to target CTGF is the monoclonal antibody pamrevlumab (FG-3019); Pamrevlumab successfully attenuated tumour growth, metastasis and angiogenesis and in a murine PDAC model [107]. Murine models of PDAC treated with the Pamrevlumab/gemcitabine combination had significantly prolonged survival compared with gemcitabine alone (median survival 29 versus 7.5 days, *p* = 0.03) [108].

### Effects of targeting the CTGF in clinical studies

A phase I study assessed the combination of pamrevlumab, gemcitabine and erlotinib in advanced PDAC patients, it showed that overall survival improved with increasing doses of pamrevlumab with a good safety profile [109]. A randomised trial compared gemcitabine/nab-paclitaxel to gemcitabine/nab paclitaxel with pamrevlumab as neoadjuvant therapies in patients with locally advanced PDAC, it revealed that the addition of pamrevlumab improved the rate of successful resection was achieved in 8 (33% in patients treated with pamrevlumab + gemcitabine/nab-paclitaxel versus 8% in patients treated and with gemcitabine/nab-paclitaxel alone, *p* = 0.1193) [110].

## Hypoxia-activated cytotoxic prodrugs

### Background

The dense desmoplastic stroma of PDAC compresses the vasculature and induces hypoxia in the tumour microenvironment [111]. This hypoxia affects the expression of many genes in carcinoma and stromal cells, contributing to many PDAC characteristics, the most important being resistance to chemotherapy [112, 113]. In addition, hypoxia promotes tumour growth, invasion and metastasis [114–116]. Hypoxia-activated cytotoxic prodrugs were theorised to have greater efficacy and minimal toxicity as they are only activated in hypoxic environments. Evofosfamide (TH-302), a hypoxia-activated prodrug; is activated to its cytotoxic form, bromo-isophospharmide, only in hypoxic conditions [117, 118].

### Effects of targeting the tumour hypoxia with hypoxia-activated prodrugs in preclinical studies

Evofosfamide improved sensitivity to radiation and gemcitabine/nab-paclitaxel in murine models of PDAC, suggesting that hypoxia is a primary contributor to chemoresistance and radioresistance [119, 120].

### Effects of targeting the tumour hypoxia with hypoxia-activated prodrugs in clinical studies

A phase III clinical trial reported that the gemcitabine/evafosfamide combination had significantly prolonged PFS compared to gemcitabine with a placebo (HR = 0.77, *p* = 0.004). However, the addition of evafosfamide did not improve overall survival [121].

## Targeting glutamine metabolism

### Background

Altered cellular metabolism is widely recognised as a hallmark of cancer. Warburg metabolism is activated in cancer cells, where oxidative phosphorylation is inhibited. Moreover, pyruvate derived from glucose is no longer the source of Krebs cycle intermediates. Instead, glutamine uptake is activated, becoming the primary cellular source of Krebs cycle intermediates via anaplerotic reactions. In addition, glutamine becomes an essential source of cancerous cell’s lipids, proteins and nucleotides, and it is essential for maintaining cellular redox status [122–124]. In PDAC, reprogramming of glutamine metabolism is mediated by oncogenic Kirsten rat sarcoma viral oncogene homolog (K-RAS), the most commonly mutated protein in PDAC [125]. Increased glutamine catabolism is a major mechanism of hypoxia and chemoresistance in PDAC cells [126]. SLC38A5, a glutamine transporter, is overexpressed in gemcitabine-resistant PDAC patients compared to gemcitabine-sensitive ones [127]. Targeting the glutamine metabolic pathway might be a promising therapeutic approach for PDAC patients.

### Effects of targeting the glutamine metabolism in preclinical studies

6-diazo-5-oxo-L-norleucine (DON) is a glutamine antagonist that inhibits many aspects of glutamine metabolism by competitively binding enzymes that utilise glutamine. It was tested for its ability to block PDAC tumour growth and metastasis using murine models. Recouvreux *et al* [129] found that cancerous cells bypass the effects of DON by increasing the availability of asparagine, suggesting that depleting asparagine by inhibiting the enzyme asparagine synthase, which utilises glutamine as a substrate, was necessary for inhibiting cellular growth. This theory is supported by the finding that combining DON with L-asparaginase, an enzyme that depletes asparagine, dramatically enhances DON's inhibitory effects in murine PDAC models [128, 129].

Sirpiglenastat (DRP-104) is a prodrug of DON; it increased the survival of murine models of PDAC when combined with trametinib (MAPK/ERK kinase inhibitor). The rationale behind combining trametinib with sirpiglenastat is that cancer cells overcome the metabolic stress caused by DRP-104 by activating the ERK pathway (ERK) signaling pathway. So, using the MEK (mitogen-activated protein kinase kinase) inhibitor trametinib reduces resistance to sirpiglenastat and enhances its effects on tumour growth [130].

## Targeting glycogen synthase kinase-3β

### Background

Glycogen synthase kinase-3β, a versatile cellular serine-threonine kinase, is involved in many physiological cellular processes, including glycogen metabolism, insulin signaling, cellular proliferation and differentiation. Although initially thought to act as a tumour suppressor by inhibiting the oncogenic Wnt/β-catenin pathway [131, 132]. It is now clear that GSK-3β plays a pro-oncogenic role in PDAC as well as other cancers. It supports the invasion, metastasis and chemoresistance of PDAC by activating several cellular pathways, including the NF-κB, CXCR4/MMP-2, RB protein/E2F pathways and many others [133–136].

Inhibiting GSK-3β may be a promising therapeutic strategy in PDAC. Several agents are available that inhibit GSK-3β, including the FDA-approved antihelminthic agent niclosamide, the experimental chemical 9-ING-41 and the most recently identified MJ34.

### Effects of targeting the GSK-3β in preclinical studies

Niclosamide showed several beneficial effects on PDAC cell lines *in vitro*. It inhibits cell proliferation, induces apoptosis through the intrinsic pathway and suppresses cell migration and invasion by antagonising. In PDAC murine models, niclosamide inhibited tumour growth and metastasis. Furthermore, niclosamide sensitises cancer cells to gemcitabine and reduces cancer immune evasion by downregulating PD-L1 expression [137].

9-ING-41 is another small-molecule inhibitor of GSK-3β. It showed therapeutic potential in preclinical models of PDAC. It may act by increasing the degradation of the protein TopBP1, which is involved in DNA repair, thus sensitising PDAC to DNA-damaging cytotoxic agents. It is currently being assessed in clinical trials for many solid tumours, including PDAC [138, 139].

MJ34 is a novel potent GSK3β inhibitor. It significantly reduces the growth and survival of human PDAC cells, mainly by inducing apoptosis in a β-catenin-dependent manner [140].

## Vitamin A analogues

### Background

In healthy individuals, PSCs exhibit a quiescent state, in which these cells store vitamin A and exhibit little synthetic activity. Once activated, PSCs acquire a secretory phenotype characterised by loss of vitamin A droplets, increased synthesis of ECM proteins (e.g., collagen and fibronectin) and enhanced proliferation and migration. Activated PSCs can be identified in tissue by their expression of the markers α-smooth muscle actin [141–143]. Activated PSCs are responsible for the dense fibrosis seen in PDAC, as it was observed that tumour cells secrete factors that activate PSCs to synthesise stromal components [144–146]. It is also observed that all-trans retinoic acid (ATRA), a vitamin A derivative, successfully reverses activation of PSCs both *in vitro* and in murine models of PDAC by altering genetic expression in these cells [147, 148].

### Effects of targeting the Vitamin A pathway in preclinical studies

ATRA binds retinoic acid receptor β expressed in PSCs, resulting in many inhibitory effects, including transcriptional repression of myosin light chain 2, which results in impaired mechanosensing function of PSCs and reduced ability of cancer cells to penetrate through the basement membrane [149–150]. ATRA also reduces collagen synthesis in PSCs by inhibition of AP-1 transcription factor [147] and inhibition of TGF-β secretion, which acts in an autocrine fashion to maintain activation of PSCs [151]. Induction of quiescence of PSCs by ATRA also resulted in the inhibition of nearby cancer cells. Froeling *et al* [152] reported that ATRA significantly reduced pro-oncogenic Wnt/β-catenin signaling, resulting in slower tumour progression *in vitro* and murine models. In addition, ATRA enhanced infiltration of PDAC by antitumour CD8+ T cells *in vitro* [153]. Kuroda *et al* [154] showed that ATRA enhanced PDAC sensitivity to gemcitabine by upregulating deoxycytidine kinase (the rate-limiting enzyme of gemcitabine activation) *in vitro*.

### Effects of targeting the Vitamin A pathway in clinical studies

Based on these broad observations, a phase I clinical trial evaluated the safety of a regimen combination of ATRA, Gemcitabine and Nab-paclitaxel for patients with PDAC, it revealed that the three-drug regimen was well tolerated and it may improve overall survival compared to Nab-paclitaxel/gemcitabine alone [155].

## Vitamin D analogues

### Background

Transcriptome studies of PSCs isolated from PDAC have shown that both quiescent and active PSCs strongly express the Vitamin D receptor (VDR); this receptor is activated by binding the active form of vitamin D (1,25-dihydroxycholecalciferol or vitamin D_3_) [156]. VDR is an intracellular receptor that acts as a master transcriptional regulator of PSCs, just like the RAR-β receptor. Activation of VDR induces quiescence of PSCs and inhibits fibrosis in PDAC [157]. Wang *et al* [158] 2015 reported that a low VDR expression in PDAC is associated with shorter survival. This may be related to the beneficial antistromal effects of vitamin D signaling [158].

### Effects of targeting the Vitamin D pathway in preclinical studies

Calcipotriol reduced the tumour-promoting activity of CAFs, reduced the expression of mucin and antagonised the pro-oncogenic Wnt/β-catenin signaling pathway *in vitro*. However, calcipotriol inhibits antitumour T cell effector functions, which could potentially weaken the patients' antitumour immune response [159–161].

Vitamin D analogs, including Calcipotriol and paricalcitol, may be beneficial as chemosensitising agents, as multiple studies found that they enhance tumour response to gemcitabine both *in vitro* and *in vivo* [157, 161, 162]. Proposed mechanisms include increased intratumoural bioavailability of cytotoxic drugs and reduced expression of mucin.

### Effects of a combination of vitamins A and D analogues

Combination therapy with the vitamins A and D analogs, 13-cis retinoic acid and 1,25-dihydroxy vitamin D3, significantly inhibits tumour invasion and the expression of MMPs in PDAC cells *in vitro* by blocking the JNK and NF-κB signaling pathways [163].

### Targeting tumour vasculature/angiogenesis

### Background

Angiogenesis, the ability to induce the growth of new blood vessels, is recognised as a hallmark of cancer. It is crucial for tumour development and progression, as tumours cannot grow beyond diffusion limitation without blood vessel formation [50]. Targeting angiogenesis is considered a potential therapeutic strategy in PDAC, as antiangiogenic drugs have succeeded in treating many solid tumours, including colorectal and renal cell carcinomas.

VEGF is a well-recognised pro-angiogenic factor that is overexpressed in many solid tumours. Its expression is associated with a higher frequency of liver metastasis and a shorter survival prognosis in patients with PDAC [164]. VEGF is secreted to act in a paracrine fashion. It binds to its receptors, vascular endothelial growth factor receptor (VEGFRs), which are receptor tyrosine kinases (RTK) that phosphorylate downstream proteins, finally promoting angiogenesis signaling.

The HGF/C-Met pathway operates by secretion of HGF in a paracrine fashion, which binds to the C-Met receptor, an RTK that phosphorylates downstream proteins. This pathway plays a vital role in tumour angiogenesis. The HGF/c-MET pathway is involved in many human physiological functions, including embryonic development and tissue repair. However, this pathway is not constitutionally active in healthy human adults except in malignant tumours. In addition to promoting angiogenesis, activation of this pathway promotes the growth and motility of PDAC cells [165]. Clinical data support the role of HFG/c-MET in the progression of PDAC. Serum levels of soluble HGF in PDAC patients are associated with disease progression [166], and high tumour expression of c-MET is associated with poor prognosis [167].

### Effects of targeting the tumour angiogenesis in preclinical studies

Bevacizumab is a VEGF-inhibiting antibody that is FDA-approved for the treatment of many solid malignancies. In a preclinical murine model of PDAC, addition of Bevacizumab and cetuximab (epidermal growth factor receptor (EGFR) inhibitory antibody) with gemcitabine/FOLOFRINOX significantly reduced pancreatic tumour weight compared to mice treated with chemotherapy alone [168].

Cabozantinib is a small-molecule tyrosine kinase inhibitor (TKI) of both c-MET and VEGFR-2; it is FDA-approved for the treatment of renal and hepatocellular carcinoma. Preclinical studies showed that cabozantinib increased the efficacy of gemcitabine, inhibited tumour growth, reduced vasculature, tumour aggressiveness, cancer stem cell population and inhibited metastasis [169]. Another c-MET inhibitor, Crizotinib, successfully shrunk tumours, enhanced survival, enhanced systemic and intratumoural bioavailability of gemcitabine and inhibited peritoneal dissemination in murine models of PDAC [170, 171]. Capmatinib is a highly selective C-Met inhibitor that has also shown efficacy in preclinical models of PDAC [172].

### Effects of targeting the tumour angiogenesis in clinical studies

A treatment regimen of Bevacizumab combined with FOLFIRINOX was tolerable in PDAC patients in a small single-arm phase I clinical trial [173]. A phase III clinical trial was conducted to compare gemcitabine/bevacizumab versus gemcitabine/placebo regimens in 602 advanced PDAC patients. It revealed that the addition of bevacizumab did not improve survival [174]. In another phase III trial, the addition of bevacizumab to the gemcitabine/erlotinib regimen resulted in increased PFS compared to gemcitabine/erlotinib alone (HR = 0.73; *p* = 0.0002). However, it failed to improve overall survival. Ramucirumab is a monoclonal antibody that targets VEGFR2, it has been investigated in clinical trials for PDAC. A phase II clinical trial was conducted to compare PFS between mFOLFIRINOX with or without Ramucirumab as first-line therapy in 82 patients with metastatic PDAC. Results showed that the overall survival rates and PFS have not been significantly impacted between the two arms of treatment [175]. The clinical efficacy of cabozantinib is being evaluated in randomised phase II studies involving many solid tumours, which include PDAC.

## Other TKIs

### Background

The role of RTKs is not confined to angiogenesis; instead, they play a crucial role in other aspects of promoting carcinogenesis, including constitutional activation of the cell cycle, reprogramming cellular metabolism and other functions. Examples of RTKs implicated in PDAC include EGFR, fibroblast growth factor receptor (FGFR), insulin-like growth factor-I receptor and VEGFR, among others. In normal cells, RTK activity is tightly regulated. Dysregulation and constitutive activation of RTKs is a common feature of many cancers, including PDAC. Many TKIs are now approved for treating many solid and hematological cancers. Therefore, targeting different types of RTKs may provide benefits in PDAC [176]. Table 4 summarises the results of targeting different RTKs in PDAC.

### Effects of targeting other tyrosine kinases in preclinical studies

Erlotinib is a small-molecule TKI that specifically targets EGFR, resulting in the inhibition of downstream K-RAS. It showed significant antitumour activity against pancreatic cancer in preclinical and early clinical evaluations when combined with gemcitabine [177, 178]. Ibrutinib is an inhibitor of Bruton's tyrosine kinase (BTK), an important tyrosine kinase that regulates intracellular signaling in B lymphocytes. The infiltration of BTK-expressing cells correlates with poor outcomes in PDAC. Ibrutinib effectively limits the growth of PDAC in both transgenic and patient-derived xenograft models of PDAC [179]. Nintedanib is a broad-spectrum TKI targeting VEGFR, PDGFR and FGFR; it is FDA-approved for treating idiopathic pulmonary fibrosis. When investigated in a murine model of PDAC, Nintedanib reduced the tumour's vascular density and collagen content and interestingly, it induced a transformative shift in the desmoplastic and immune landscape, increasing the number of antitumour CD8+ T cells and reducing tumour-protecting FOXP3+ T cells. Thus, it enhanced the antitumour effects of anti-PD-1 immunotherapy [180, 181]. Additionally, Nintedanib enhanced the activity of another form of immunotherapy: chimeric antigen receptor natural killer cells in a xenograft murine model [182]. Sunitinib, another TKI, has been shown to reduce tumour growth, endothelial cell proliferation, fibroblast proliferation and median survival in murine xenograft models, mainly when used in combination therapies [183].

The FGF signaling pathway, regulated by 18 growth factors and four FGFRs tyrosine kinases, is crucial for cell growth, development and differentiation. Disruptions in this pathway are linked to various malignancies, including PDAC [184]. In PDAC, the activation of FGF/FGFR signaling is essential for disease progression and interacts with other pathways in pancreatic cancer, underscoring the complexity of FGF signaling. The therapeutic potential of targeting this pathway is significant, as evidenced by using TKIs to treat cancers driven by aberrant FGF signaling [185–187].

A study investigated a combination of gemcitabine with BGJ398 (infigratinib), a pan-FGFR inhibitor, revealing potential mechanisms for improved efficacy [188]. Beyond this specific interaction, FGFR inhibitors offer broader benefits, potentially improving treatment outcomes for many PDAC treatment strategies in several ways [189].

### Effects of targeting other tyrosine kinases in clinical studies

A multicenter randomised Phase III trial was conducted to compare the therapeutic effects of gemcitabine plus erlotinib compared to gemcitabine alone in patients following resection of pancreatic cancer. Erlotinib successfully improved PFS and overall survival. It is thought that erlotinib is particularly effective in tumours with activated EGFR and wild-type K-RAS. This is not the case in PDAC, as in most cases, K-RAS is constitutionally activated downstream of EGFR [190]. In the phase III RESOLVE trial, the combination of ibrutinib with nab-paclitaxel/gemcitabine was tested in treatment-naïve patients with metastatic PDAC. Unfortunately, the combination did not improve survival [191]. A phase II single-arm trial evaluated dasatinib as the initial treatment for patients with metastatic PDAC. The findings indicated that dasatinib alo Dasatinib and masitinib are oral TKIs that target multiple proteins, including BCR-ABL, c-Src, c-KIT, platelet-derived growth factor receptor β and EphA2. They are FDA-approved for the treatment of chronic myeloid leukaemia, gastrointestinal stromal tumours and c-KIT mutated melanoma, among other tumours. The findings indicated that dasatinib alone did not demonstrate clinical effectiveness in treating metastatic PDAC [192]. Despite increased toxicity and manageable side effects, masitinib significantly increased overall survival when combined with gemcitabine compared to gemcitabine alone. This finding was observed in a randomised, placebo-controlled phase III trial [193].

## Conclusion and future perspectives

Targeting the dense fibrotic stroma of PDAC offers a multipronged therapeutic strategy. While many anti-stromal therapies have yielded mixed results in clinical trials, the underlying principle of improving intratumoural drug delivery and modulating the tumour microenvironment remains highly promising.

A key area requiring further investigation is the role of HA and its modulation via hyaluronidase. The MORPHEUS PDAC trial, while demonstrating similar overall survival between standard chemotherapy and the atezolizumab/PEGPH20 combination, highlighted a potentially crucial subgroup of patients. In patients with high HA content, atezolizumab plus PEGPH20 demonstrated superior overall survival compared to standard therapy. The HALO 202 trial also demonstrated that with high tumour HA content had a greater benefit from the addition of PEGPH20. These findings suggest that the efficacy of hyaluronidase-based therapies is dependent on the tumour's HA content, a factor that should be considered for future clinical trials. Careful comparison of outcomes across different HA strata will provide crucial insights into the optimal use of these agents and potentially lead to more personalised approaches to PDAC treatment.

Given the success of atezolizumab/hyaluronidase combinations in enhancing antitumour immune responses in patients with other solid tumours, including non-small-cell lung carcinoma, hepatocellular carcinoma, melanoma and other tumours [194]. Further investigation is warranted to explore the mechanisms by which hyaluronidase enhances the efficacy of immune checkpoint inhibitors, which may include improved intratumoural bioavailability of these agents, especially considering their high molecular weight as monoclonal antibodies or elimination of potential immunosuppressive effects of HA. Research to identify biomarkers whose expression may predict response to hyaluronidase, which may include HA, PD-L1 and enzymes involved in HA metabolism is warranted. Other innovative strategies to enhance drug delivery to the tumour, including the use of microbial vectors and antibody-drug conjugates, remain largely unexplored in PDAC and should be investigated further.

The role of SHh pathway in PDAC is controversial. While Rhim *et al* [10] found that SHh pathway actually restricts tumour growth in murine models, High expression of SHh pathway proteins in patients’ samples is predictive of a bad prognosis. Further research is warranted to understand the context-dependent functions of the SHh pathway. Although the majority of clinical trials did not demonstrate any advantage of including SHh inhibitors into standard cytotoxic chemotherapy, most of these trials had small sample sizes and employed a single-arm design. More comprehensive, large controlled clinical trials are warranted to determine the true effects of SHh pathway in PDAC. The therapeutic potential of SHh inhibitors other than cyclopamine derivatives remain underexplored.

The trials conducted by Murphy *et al* [86] and Picozzi *et al* [109, 110] highlight the promising potential of antistromal therapies as neoadjuvant treatments to downstage the tumour in patients with locally advanced PDAC and facilitate surgical resection. Most trials that evaluated the various antistromal therapies were conducted on patients with metastatic disease, leaving their potential benefits as neoadjuvant therapies largely unexplored. As PDAC is associated with high recurrence rates after surgical resection, further research is needed to optimise the best strategies of neoadjuvant therapy, aiming to reduce recurrence rates and improve survival. Considering the well-defined role of MMPs in cancer invasion, we think that matrix metalloproteinase inhibitors might be particularly beneficial in this setting.

Considering the promising results from targeting CTGF, further research should be directed towards identifying more growth factors that are secreted from PDAC’s CAF, which may play an important role in tumour-stroma interactions that support tumour growth, invasion, metastasis and chemoresistance.

The findings on the effects of vitamin A and nintedanib in enhancing tumour infiltration by cytotoxic T cells are particularly promising. These insights suggest that targeting the stromal components of PDAC may significantly improve the efficacy of immune checkpoint inhibitors in PDAC without microsatellite instability. Further research should focus on elucidating the immune microenvironment of PDAC, with particular emphasis on understanding how the tumour stroma contributes to immune evasion. Efforts should be directed towards developing strategies to enhance the efficacy of immunotherapies in PDAC.

Given the modest success of erlotinib in PDAC and because the most common driving mutation in human PDAC in KRAS G12D mutation, we think that targeting the EFGR-KRAS-MAPK pathway might be a successful strategy for treating PDAC. While sotorasib and adagarsib, the only commercially available KRAS inhibitors, have demonstrated success in the treatment of solid tumours, including non-small cell lung carcinoma and colorectal carcinoma, they only inhibit K-RAS with G12C mutation. MTRX1133 is an experimental inhibitor of KRAS with G12D mutation, it might become the first breakthrough therapy for PDAC [195]. As loss-of-function mutations in the CDKN2A gene, which codes for p16, an important cyclin-dependent kinase inhibitor, are common in PDAC [196], we think that treatment with cyclin-dependent kinase inhibitors (e.g., palbociclib) might also be a promising strategy.

Finally, most trials evaluating antistromal therapies in PDAC were conducted in a small number of patients or utilised a single-arm design. More comprehensive controlled clinical trials are needed to understand the true effect of these agents.

## Conflicts of interest

The authors have no conflicts of interest to disclose.

## Funding

This study was not funded. There are no sources of funding.

## Figures and Tables

**Table 1.** Strategies to target HA in PDAC.

Strategy	Agents used	Observed effects
Systemic depletion of HA by enzymatic digestion.	PEGPH20.	Enhanced delivery of multiple drugs and immune cell infiltration [14, 18, 19, 22, 23].
Targeted depletion of HA by enzymatic digestion.	Microbial vectors expressing hyaluronidase.	Enhanced drug delivery [24].
Inhibition of HA synthesis.	4-MU	Inhibition of cellular migration and enhanced immune cell infiltration [26, 27].

**Table 2.** Agents that target SHh pathway and their effect on PDAC.

Agent	Mechanism of action	Observed effects
Cyclopamine and its derivates: vismodegib, sonidegib, glasdegib and saridegib.	Inhibition of SMO signaling transducer.	Cyclopamine inhibited tumour growth [39] Vismodegib, sonidegib, and Saridegib induced clinical responses in patient when combined with gemcitabine/ FOLFIRINOX [56–59]
Itraconazole	Inhibition of SMO signaling transducer.	Inhibition of tumour growth and epithelial-to-mesenchymal transition [45, 46]
Oxy169	Inhibition of SHh pathway downstream of SOM	Inhibition of PDAC growth [49]
SB-T-1216 (taxane derivative).	Precise mechanism unknown.	Potent inhibition of SHh pathway [34]
n-Butylidenephthalide	Inhibition of DNMT1, hypomethylation of PTCHD4 gene and increasing its expression.	Growth inhibiting and induction of apoptosis [54]

**Table 3.** Various stromal components in PDAC, agents that target them and observed effects of targeting them.

Target	Agents	Observed effects
SPARC	Nab-paclitaxel.	Clinical efficacy [73]
AT II	ACEis, ARBs.	Inhibiting angiogenesis and enhancing intratumoural drug bioavailability [76, 79–83] Successful tumour downstaging in combination with gemcitabine [86].
MMPs.	Marimastat, tanomastat.	Preliminary evidence of clinical efficacy [95, 97].
Glutamine metabolism.	DON, Sirpiglenastat, L-asparaginase.	Inhibition of tumour growth *in vitro* [130]
Glycogen synthase kinase - 3β	Niclosamide, 9-ING-41, MJ34.	Sensitisation to chemotherapy and enhancement of antitumour immune response [137–139].
CTGF	Pamrevlumab	Inhibition of tumour growth, metastasis, and angiogenesis [107]
Activated PSCs	Vitamin A analogs (ATRA). Vitamin D analogs (calcipotriol, paricalcitol).	Enhancement of sensitivity to gemcitabine [154, 157, 161, 162]. Inhibition of tumour invasion and metastasis [149, 150]
Tumour vasculature, VEGF pathway, HGF/C-met pathway.	Anti-angiogenic monoclonal antibodies (bevacizumab), small molecule TKIs (cabozantinib, Crizotinib).	Inhibition of tumour growth and metastasis [168, 170]

**Table 4.** Different TKIs, their target and their effects on PDAC.

TKI	Target	Effects on PDAC
Cabozantinib	VEFGR2, C-Met	Inhibition of angiogenesis. [169]
Crizotinib	C-MET	Inhibition of angiogenesis, sensitising tumour to gemcitabine and inhibition of metastasis [170, 171]
Erlotinib	EGFR	Clinical efficacy in treatment of PDAC [190]
Ibrutinib	BTK of B lymphocytes.	Inhibiting tumour growth [179]
Nintedanib	Broad spectrum TKI	Enhancement of immune cell infiltration and sensitising tumours t immune therapy [180, 181]
Sunitinib	Board spectrum TKI	Inhibiting tumour growth [183]
Dasatinib, masitinib	Board spectrum TKI	Inhibiting tumour growth [192, 193]
Infigratinib	FGFR	Inhibiting tumour growth [188]

## References

[1] Klein AP (2021). Pancreatic cancer epidemiology: understanding the role of lifestyle and inherited risk factors. Nat Rev Gastroenterol Hepatol.

[2] Siegel RL, Giaquinto AN, Jemal A (2024). Cancer statistics, 2024. CA Cancer J Clin.

[3] Ryan DP, Hong TS, Bardeesy N (2014). Pancreatic adenocarcinoma. N Engl J Med.

[4] Kamisawa T, Wood LD, Itoi T (2016). Pancreatic cancer. Lancet Lond Engl.

[5] Conroy T (2018). FOLFIRINOX or gemcitabine as adjuvant therapy for pancreatic cancer. N Engl J Med.

[6] Leppänen J (2019). Tenascin C, fibronectin, and tumor-stroma ratio in pancreatic ductal adenocarcinoma. Pancreas.

[7] Neesse A (2011). Stromal biology and therapy in pancreatic cancer. Gut.

[8] Dougan SK (2017). The pancreatic cancer microenvironment. Cancer J Sudbury Mass.

[9] Özdemir BC (2014). Depletion of carcinoma-associated fibroblasts and fibrosis induces immunosuppression and accelerates pancreas cancer with reduced survival. Cancer Cell.

[10] Rhim AD (2014). Stromal elements act to restrain, rather than support, pancreatic ductal adenocarcinoma. Cancer Cell.

[11] Knudson CB, Knudson W (1993). Hyaluronan-binding proteins in development, tissue homeostasis, and disease. FASEB J.

[12] Theocharis AD, Tsara ME, Papageorgacopoulou N (2000). Pancreatic carcinoma is characterized by elevated content of hyaluronan and chondroitin sulfate with altered disaccharide composition. Biochim Biophys Acta.

[13] Kim PK (2021). Hyaluronic acid fuels pancreatic cancer cell growth. eLife.

[14] Thompson CB (2010). Enzymatic depletion of tumor hyaluronan induces antitumor responses in preclinical animal models. Mol Cancer Ther.

[15] Cheng XB, Kohi S, Koga A (2016). Hyaluronan stimulates pancreatic cancer cell motility. Oncotarget.

[16] Koltai T, Reshkin SJ, Carvalho TMA (2021). Targeting the stromal pro-tumoral hyaluronan-CD44 pathway in pancreatic cancer. Int J Mol Sci.

[17] Kultti A (2014). Accumulation of extracellular hyaluronan by hyaluronan synthase 3 promotes tumor growth and modulates the pancreatic cancer microenvironment. BioMed Res Int.

[18] Provenzano PP, Cuevas C, Chang AE (2012). Enzymatic targeting of the stroma ablates physical barriers to treatment of pancreatic ductal adenocarcinoma. Cancer Cell.

[19] Jacobetz MA (2013). Hyaluronan impairs vascular function and drug delivery in a mouse model of pancreatic cancer. Gut.

[20] Provenzano PP, Hingorani SR (2013). Hyaluronan, fluid pressure, and stromal resistance in pancreas cancer. Br J Cancer.

[21] Whatcott CJ (2015). Desmoplasia in primary tumors and metastatic lesions of pancreatic cancer. Clin Cancer Res.

[22] Minchinton AI, Tannock IF (2006). Drug penetration in solid tumours. Nat Rev Cancer.

[23] Blair AB (2022). Dual stromal targeting sensitizes pancreatic adenocarcinoma for anti-PD-1 therapy: dual stromal targeting in pancreatic cancer. Gastroenterology.

[24] Ebelt ND, Zuniga E, Passi KB (2020). Hyaluronidase-expressing Salmonella effectively targets tumor-associated hyaluronic acid in pancreatic ductal adenocarcinoma. Mol Cancer Ther.

[25] Bazan-Peregrino M (2021). VCN-01 disrupts pancreatic cancer stroma and exerts antitumor effects. J Immunother Cancer.

[26] Kudo Y, Kohi S, Hirata K (2019). Hyaluronan activated-metabolism phenotype (HAMP) in pancreatic ductal adenocarcinoma. Oncotarget.

[27] Suto A (2019). Increase of tumor infiltrating γδ T-cells in pancreatic ductal adenocarcinoma through remodeling of the extracellular matrix by a hyaluronan synthesis suppressor, 4-methylumbelliferone. Pancreas.

[28] Hingorani SR (2016). Phase Ib study of PEGylated recombinant human hyaluronidase and gemcitabine in patients with advanced pancreatic cancer. Clin Cancer Res.

[29] Hingorani SR (2018). HALO 202: randomized phase II study of PEGPH20 plus nab-paclitaxel/gemcitabine versus nab-paclitaxel/gemcitabine in patients with untreated, metastatic pancreatic ductal adenocarcinoma. J Clin Oncol.

[30] Hingorani SR (2015). High response rate and PFS with PEGPH20 added to nab-paclitaxel/gemcitabine in stage IV previously untreated pancreatic cancer patients with high-HA tumors: interim results of a randomized phase II study. J Clin Oncol.

[31] Cutsem EV (2020). Randomized phase III trial of pegvorhyaluronidase Alfa with nab-paclitaxel plus gemcitabine for patients with hyaluronan-high metastatic pancreatic adenocarcinoma. J Clin Oncol.

[32] Ramanathan RK (2019). Phase IB/II randomized study of FOLFIRINOX plus pegylated recombinant human hyaluronidase versus FOLFIRINOX alone in patients with metastatic pancreatic adenocarcinoma: SWOG S1313. J Clin Oncol.

[33] Ko AH (2023). Atezolizumab plus PEGPH20 versus chemotherapy in advanced pancreatic ductal adenocarcinoma and gastric cancer: MORPHEUS phase Ib/II umbrella randomized study platform. Oncologist.

[34] Mohelnikova-Duchonova B (2017). Hedgehog pathway overexpression in pancreatic cancer is abrogated by new-generation taxoid SB-T-1216. Pharmacogenomics J.

[35] Tian H (2009). Hedgehog signaling is restricted to the stromal compartment during pancreatic carcinogenesis. Proc Natl Acad Sci U S A.

[36] Bailey JM (2008). Sonic hedgehog promotes desmoplasia in pancreatic cancer. Clin Cancer Res.

[37] Yauch RL (2008). A paracrine requirement for hedgehog signalling in cancer. Nature.

[38] Maréchal R (2015). Sonic hedgehog and Gli1 expression predict outcome in resected pancreatic adenocarcinoma. Clin Cancer Res.

[39] Zhang B (2016). Cyclopamine disrupts tumor extracellular matrix and improves the distribution and efficacy of nanotherapeutics in pancreatic cancer. Biomaterials.

[40] Olive KP (2009). Inhibition of Hedgehog signaling enhances delivery of chemotherapy in a mouse model of pancreatic cancer. Science.

[41] Kim J (2013). Itraconazole and arsenic trioxide inhibit hedgehog pathway activation and tumor growth associated with acquired resistance to smoothened antagonists. Cancer Cell.

[42] Liu M, Liang G, Zheng H (2019). Triazoles bind the C-terminal domain of SMO: Illustration by docking and molecular dynamics simulations the binding between SMO and triazoles. Life Sci.

[43] Ban L (2020). Anti-fungal drug itraconazole exerts anti-cancer effects in oral squamous cell carcinoma via suppressing Hedgehog pathway. Life Sci.

[44] Wang X (2017). Anti-proliferation of breast cancer cells with itraconazole: hedgehog pathway inhibition induces apoptosis and autophagic cell death. Cancer Lett.

[45] Liu Z (2019). Paclitaxel and itraconazole co-encapsulated micelle prolongs the survival of spontaneous LSL-KrasG12D/+, LSL-Trp53R172H/+, Pdx-1-Cre genetically engineered mouse model of pancreatic cancer. Adv Ther.

[46] Chen K (2018). Itraconazole inhibits invasion and migration of pancreatic cancer cells by suppressing TGF-β/SMAD2/3 signaling. Oncol Rep.

[47] Nolan-Stevaux O (2009). GLI1 is regulated through Smoothened-independent mechanisms in neoplastic pancreatic ducts and mediates PDAC cell survival and transformation. Genes Dev.

[48] Rajurkar M (2012). The activity of Gli transcription factors is essential for Kras-induced pancreatic tumorigenesis. Proc Natl Acad Sci U S A.

[49] Wang F, Stappenbeck F, Parhami F (2019). Inhibition of hedgehog signaling in fibroblasts, pancreatic, and lung tumor cells by Oxy186, an oxysterol analogue with drug-like properties. Cells.

[50] Hanahan D (2022). Hallmarks of cancer: new dimensions. Cancer Discov.

[51] Li A, Omura N, Hong SM (2010). Pancreatic cancer DNMT1 expression and sensitivity to DNMT1 inhibitors. Cancer Biol Ther.

[52] Sato N, Fukushima N, Hruban RH (2008). CpG island methylation profile of pancreatic intraepithelial neoplasia. Mod Pathol.

[53] Bhattacharyya S (2017). Altered hydroxymethylation is seen at regulatory regions in pancreatic cancer and regulates oncogenic pathways. Genome Res.

[54] Huang MH (2019). Epigenetic targeting DNMT1 of pancreatic ductal adenocarcinoma using interstitial control release biodegrading polymer reduced tumor growth through hedgehog pathway inhibition. Pharmacol Res.

[55] Li H (2020). TET1 downregulates epithelial-mesenchymal transition and chemoresistance in PDAC by demethylating CHL1 to inhibit the Hedgehog signaling pathway. Oncogene.

[56] Kim EJ (2014). Pilot clinical trial of hedgehog pathway inhibitor GDC-0449 (vismodegib) in combination with gemcitabine in patients with metastatic pancreatic adenocarcinoma. Clin Cancer Res.

[57] Catenacci DVT (2015). Randomized phase Ib/II study of gemcitabine plus placebo or vismodegib, a hedgehog pathway inhibitor, in patients with metastatic pancreatic cancer. J Clin Oncol.

[58] Pijnappel EN (2021). Phase I/II study of LDE225 in combination with gemcitabine and nab-paclitaxel in patients with metastatic pancreatic cancer. Cancers.

[59] Clark JW (2021). A phase 1b clinical trial of LDE225 (Sonidegib) in combination with fluorouracil, leucovorin, oxaliplatin, and irinotecan (FOLFIRINOX) in previously untreated locally advanced or metastatic pancreatic adenocarcinoma. Ann Pancreat Cancer.

[60] Stephenson J (2011). The safety of IPI-926, a novel hedgehog pathway inhibitor, in combination with gemcitabine in patients (pts) with metastatic pancreatic cancer. J Clin Oncol.

[61] Jimeno A (2013). Phase I study of the Hedgehog pathway inhibitor IPI-926 in adult patients with solid tumors. Clin Cancer Res.

[62] Ko AH (2016). A phase I study of FOLFIRINOX Plus IPI-926, a hedgehog pathway inhibitor, for advanced pancreatic adenocarcinoma. Pancreas.

[63] Lane TF, Sage EH (1994). The biology of SPARC, a protein that modulates cell-matrix interactions. FASEB J.

[64] Bradshaw AD, Sage EH (2001). SPARC, a matricellular protein that functions in cellular differentiation and tissue response to injury. J Clin Invest.

[65] Gundewar C, Sasor A, Hilmersson KS (2015). The role of SPARC expression in pancreatic cancer progression and patient survival. Scand J Gastroenterol.

[66] Guweidhi A (2005). Osteonectin influences growth and invasion of pancreatic cancer cells. Ann Surg.

[67] Infante JR (2007). Peritumoral fibroblast SPARC expression and patient outcome with resectable pancreatic adenocarcinoma. J Clin Oncol.

[68] Guarneri V, Dieci MV, Conte P (2012). Enhancing intracellular taxane delivery: current role and perspectives of nanoparticle albumin-bound paclitaxel in the treatment of advanced breast cancer. Expert Opin Pharmacother.

[69] Fukahori M (2021). A phase II study of gemcitabine plus nab-paclitaxel as first-line therapy for locally advanced pancreatic cancer. Medicine.

[70] Passacantilli I, Panzeri V, Terracciano F (2018). Co-treatment with gemcitabine and nab-paclitaxel exerts additive effects on pancreatic cancer cell death. Oncol Rep.

[71] Philip PA (2020). Nab-paclitaxel plus gemcitabine in patients with locally advanced pancreatic cancer (LAPACT): a multicentre, open-label phase 2 study. Lancet Gastroenterol Hepatol.

[72] Pignon F (2021). Efficacy and tolerance of gemcitabine and nab-paclitaxel in elderly patients with advanced pancreatic ductal adenocarcinoma. Pancreatology.

[73] Von Hoff DD (2013). Randomized phase III study of weekly nab-paclitaxel plus gemcitabine versus gemcitabine alone in patients with metastatic adenocarcinoma of the pancreas (MPACT). J Clin Oncol.

[74] Hidalgo M (2015). SPARC expression did not predict efficacy of nab-paclitaxel plus gemcitabine or gemcitabine alone for metastatic pancreatic cancer in an exploratory analysis of the phase III MPACT trial. Clin Cancer Res.

[75] Hama K (2004). Angiotensin II stimulates DNA synthesis of rat pancreatic stellate cells by activating ERK through EGF receptor transactivation. Biochem Biophys Res Commun.

[76] Masamune A (2013). The angiotensin II type I receptor blocker olmesartan inhibits the growth of pancreatic cancer by targeting stellate cell activities in mice. Scand J Gastroenterol.

[77] Angiotensin II type 2 receptor signaling significantly attenuates growth of murine pancreatic carcinoma grafts in syngeneic mice. BMC Cancer.

[78] Pallasch FB, Schumacher U (2020). Angiotensin inhibition, TGF-β and EMT in Cancer. Cancers.

[79] Chauhan VP (2013). Angiotensin inhibition enhances drug delivery and potentiates chemotherapy by decompressing tumour blood vessels. Nat Commun.

[80] Diop-Frimpong B, Chauhan VP, Krane S (2011). Losartan inhibits collagen I synthesis and improves the distribution and efficacy of nanotherapeutics in tumors. Proc Natl Acad Sci U S A.

[81] Kpeglo D, Haddrick M, Knowles MA (2024). Modelling and breaking down the biophysical barriers to drug delivery in pancreatic cancer. Lab Chip.

[82] Kumar V (2016). Noninvasive assessment of losartan-induced increase in functional microvasculature and drug delivery in pancreatic ductal adenocarcinoma. Transl Oncol.

[83] Noguchi R (2009). Synergistic inhibitory effect of gemcitabine and angiotensin type-1 receptor blocker, losartan, on murine pancreatic tumor growth via anti-angiogenic activities. Oncol Rep.

[84] Keith SW (2022). Angiotensin blockade therapy and survival in pancreatic cancer: a population study. BMC Cancer.

[85] Kasi A (2021). Association of losartan with outcomes in metastatic pancreatic cancer patients treated with chemotherapy. J Clin Transl Res.

[86] Murphy JE (2019). Total neoadjuvant therapy with FOLFIRINOX in combination with losartan followed by chemoradiotherapy for locally advanced pancreatic cancer: a phase 2 clinical trial. JAMA Oncol.

[87] Nakai Y (2013). A multicenter phase II trial of gemcitabine and candesartan combination therapy in patients with advanced pancreatic cancer: GECA2. Invest New Drugs.

[88] Jones L, Ghaneh P, Humphreys M (1999). The matrix metalloproteinases and their inhibitors in the treatment of pancreatic cancer. Ann N Y Acad Sci.

[89] Drifka CR, Loeffler AG, Esquibel CR (2016). Human pancreatic stellate cells modulate 3D collagen alignment to promote the migration of pancreatic ductal adenocarcinoma cells. Biomed Microdevices.

[90] Koikawa K (2018). Basement membrane destruction by pancreatic stellate cells leads to local invasion in pancreatic ductal adenocarcinoma. Cancer Lett.

[91] Li X (2014). Sonic hedgehog paracrine signaling activates stromal cells to promote perineural invasion in pancreatic cancer. Clin Cancer Res.

[92] Tjomsland V, Pomianowska E, Aasrum M (2016). Profile of MMP and TIMP expression in human pancreatic stellate cells: regulation by IL-1α and TGFβ and implications for migration of pancreatic cancer cells. Neoplasia N Y N.

[93] Xie J (2021). Identification of potential diagnostic biomarkers in MMPs for pancreatic carcinoma. Medicine.

[94] Bramhall SR, Neoptolemos JP, Stamp GW (1997). Imbalance of expression of matrix metalloproteinases (MMPs) and tissue inhibitors of the matrix metalloproteinases (TIMPs) in human pancreatic carcinoma. J Pathol.

[95] Bramhall SR, Rosemurgy A, Brown PD (2001). Marimastat as first-line therapy for patients with unresectable pancreatic cancer: a randomized trial. J Clin Oncol.

[96] Bramhall SR, Schulz J, Nemunaitis J (2002). A double-blind placebo-controlled, randomised study comparing gemcitabine and marimastat with gemcitabine and placebo as first line therapy in patients with advanced pancreatic cancer. Br J Cancer.

[97] Moore MJ (2003). Comparison of gemcitabine versus the matrix metalloproteinase inhibitor BAY 12–9566 in patients with advanced or metastatic adenocarcinoma of the pancreas: a phase III trial of the National Cancer Institute of Canada Clinical Trials Group. J Clin Oncol.

[98] Lv Q, Wang J, Xu C (2020). Pirfenidone alleviates pulmonary fibrosis in vitro and in vivo through regulating Wnt/GSK-3β/β-catenin and TGF-β1/Smad2/3 signaling pathways. Mol Med Camb Mass.

[99] Shi Q (2011). In vitro effects of pirfenidone on cardiac fibroblasts: proliferation, myofibroblast differentiation, migration and cytokine secretion. PLoS One.

[100] Liu X, Yang Y, Guo X (2017). The antiangiogenesis effect of pirfenidone in wound healing in vitro. J Ocul Pharmacol Ther.

[101] Usugi E (2019). Antifibrotic agent pirfenidone suppresses proliferation of human pancreatic cancer cells by inducing G0/G1 cell cycle arrest. Pharmacology.

[102] Lei Y (2024). Pirfenidone alleviates fibrosis by acting on tumour-stroma interplay in pancreatic cancer. Br J Cancer.

[103] Zhang Y (2024). CAFs homologous biomimetic liposome bearing BET inhibitor and pirfenidone synergistically promoting antitumor efficacy in pancreatic ductal adenocarcinoma. Adv Sci.

[104] Kozono S (2013). Pirfenidone inhibits pancreatic cancer desmoplasia by regulating stellate cells. Cancer Res.

[105] Charrier A, Brigstock DR (2013). Regulation of pancreatic function by connective tissue growth factor (CTGF, CCN2). Cytokine Growth Factor Rev.

[106] Bennewith KL (2009). The role of tumor cell-derived connective tissue growth factor (CTGF/CCN2) in pancreatic tumor growth. Cancer Res.

[107] Aikawa T, Gunn J, Spong SM (2006). Connective tissue growth factor-specific antibody attenuates tumor growth, metastasis, and angiogenesis in an orthotopic mouse model of pancreatic cancer. Mol Cancer Ther.

[108] Neesse A (2013). CTGF antagonism with mAb FG-3019 enhances chemotherapy response without increasing drug delivery in murine ductal pancreas cancer. Proc Natl Acad Sci U S A.

[109] Picozzi VJ (2014). FG-3019, a human monoclonal antibody to connective tissue growth factor (CTGF), with gemcitabine/erlotinib (G/E) in patients with locally advanced or metastatic pancreatic ductal adenocarcinoma (PDAC). J Clin Oncol.

[110] Picozzi V (2020). Gemcitabine/nab-paclitaxel with pamrevlumab: a novel drug combination and trial design for the treatment of locally advanced pancreatic cancer. ESMO Open.

[111] Erkan M (2009). Cancer-stellate cell interactions perpetuate the hypoxia-fibrosis cycle in pancreatic ductal adenocarcinoma. Neoplasia N Y N.

[112] Schober M (2014). Desmoplasia and chemoresistance in pancreatic cancer. Cancers.

[113] Shah VM, Sheppard BC, Sears RC (2020). Hypoxia: friend or foe for drug delivery in pancreatic cancer. Cancer Lett.

[114] Bailey KM (2014). Evaluation of the ‘steal’ phenomenon on the efficacy of hypoxia activated prodrug TH-302 in pancreatic cancer. PLoS One.

[115] Guillaumond F, Iovanna JL, Vasseur S (2014). Pancreatic tumor cell metabolism: focus on glycolysis and its connected metabolic pathways. Arch Biochem Biophys.

[116] Li H (2021). Hypoxia promotes the metastasis of pancreatic cancer through regulating NOX4/KDM5A-mediated histone methylation modification changes in a HIF1A-independent manner. Clin Epigenetics.

[117] Duan JX (2008). Potent and highly selective hypoxia-activated achiral phosphoramidate mustards as anticancer drugs. J Med Chem.

[118] Weiss GJ (2011). Phase 1 study of the safety, tolerability, and pharmacokinetics of TH-302, a hypoxia-activated prodrug, in patients with advanced solid malignancies. Clin Cancer Res.

[119] Hajj C (2017). A combination of radiation and the hypoxia-activated prodrug evofosfamide (TH-302) is efficacious against a human orthotopic pancreatic tumor model. Transl Oncol.

[120] Sun JD (2015). Efficacy and safety of the hypoxia-activated prodrug TH-302 in combination with gemcitabine and nab-paclitaxel in human tumor xenograft models of pancreatic cancer. Cancer Biol Ther.

[121] Van Cutsem E (2016). MAESTRO: a randomized, double-blind phase III study of evofosfamide (Evo) in combination with gemcitabine (Gem) in previously untreated patients (pts) with metastatic or locally advanced unresectable pancreatic ductal adenocarcinoma (PDAC).

[122] Lyssiotis CA, Son J, Cantley LC (2013). Pancreatic cancers rely on a novel glutamine metabolism pathway to maintain redox balance. Cell Cycle.

[123] Metallo CM (2011). Reductive glutamine metabolism by IDH1 mediates lipogenesis under hypoxia. Nature.

[124] Yoo HC (2020). A variant of SLC1A5 is a mitochondrial glutamine transporter for metabolic reprogramming in cancer cells. Cell Metab.

[125] Son J (2013). Glutamine supports pancreatic cancer growth through a Kras-regulated metabolic pathway. Nature.

[126] Park SJ (2023). Enhanced glutaminolysis drives hypoxia-induced chemoresistance in pancreatic cancer. Cancer Res.

[127] Kim MJ (2023). SLC38A5 modulates ferroptosis to overcome gemcitabine resistance in pancreatic cancer. Cells.

[128] Biancur DE (2017). Compensatory metabolic networks in pancreatic cancers upon perturbation of glutamine metabolism. Nat Commun.

[129] Recouvreux MV (2024). Glutamine mimicry suppresses tumor progression through asparagine metabolism in pancreatic ductal adenocarcinoma. Nat Cancer.

[130] Encarnación-Rosado J (2024). Targeting pancreatic cancer metabolic dependencies through glutamine antagonism. Nat Cancer.

[131] Nagini S, Sophia J, Mishra R (2019). Glycogen synthase kinases: moonlighting proteins with theranostic potential in cancer. Semin Cancer Biol.

[132] Xue W, Yang L, Chen C (2024). Wnt/β-catenin-driven EMT regulation in human cancers. Cell Mol Life Sci CMLS.

[133] Kitano A (2013). Aberrant glycogen synthase kinase 3β is involved in pancreatic cancer cell invasion and resistance to therapy. PLoS One.

[134] Ougolkov AV, Fernandez-Zapico ME, Savoy DN (2005). Glycogen synthase kinase-3beta participates in nuclear factor kappaB-mediated gene transcription and cell survival in pancreatic cancer cells. Cancer Res.

[135] Uehara M (2020). Glycogen synthase kinase-3β participates in acquired resistance to gemcitabine in pancreatic cancer. Cancer Sci.

[136] Ying X (2015). GSK3β mediates pancreatic cancer cell invasion in vitro via the CXCR4/MMP-2 Pathway. Cancer Cell Int.

[137] Guo Y (2022). The anthelmintic drug niclosamide induces GSK-β-mediated β-catenin degradation to potentiate gemcitabine activity, reduce immune evasion ability and suppress pancreatic cancer progression. Cell Death Dis.

[138] Ding L (2019). Glycogen synthase kinase-3 inhibition sensitizes pancreatic cancer cells to chemotherapy by abrogating the topBP1/ATR-mediated DNA damage response. Clin Cancer Res.

[139] Park R, Coveler AL, Cavalcante L (2021). GSK-3β in pancreatic cancer: spotlight on 9-ING-41, its therapeutic potential and immune modulatory properties. Biology.

[140] Ayaz MO (2024). Identification of a novel GSK3β inhibitor involved in abrogating KRas dependent pancreatic tumors in Wnt/beta-catenin and NF-kB dependent manner. Life Sci.

[141] Apte MV (1998). Periacinar stellate shaped cells in rat pancreas: identification, isolation, and culture. Gut.

[142] Bachem MG (1998). Identification, culture, and characterization of pancreatic stellate cells in rats and humans. Gastroenterology.

[143] Wehr AY, Furth EE, Sangar V (2011). Analysis of the human pancreatic stellate cell secreted proteome. Pancreas.

[144] Apte MV (2004). Desmoplastic reaction in pancreatic cancer: role of pancreatic stellate cells. Pancreas.

[145] Bachem MG (2005). Pancreatic carcinoma cells induce fibrosis by stimulating proliferation and matrix synthesis of stellate cells. Gastroenterology.

[146] Vonlaufen A (2008). Pancreatic stellate cells: partners in crime with pancreatic cancer cells. Cancer Res.

[147] Jaster R (2003). Regulation of pancreatic stellate cell function in vitro: Biological and molecular effects of all-trans retinoic acid. Biochem Pharmacol.

[148] McCarroll JA, Phillips PA, Santucci N (2006). Vitamin A inhibits pancreatic stellate cell activation: implications for treatment of pancreatic fibrosis. Gut.

[149] Chronopoulos A (2016). ATRA mechanically reprograms pancreatic stellate cells to suppress matrix remodelling and inhibit cancer cell invasion. Nat Commun.

[150] Matellan C (2023). Retinoic acid receptor β modulates mechanosensing and invasion in pancreatic cancer cells via myosin light chain. Oncogenesis.

[151] Sarper M, Cortes E, Lieberthal TJ (2016). ATRA modulates mechanical activation of TGF-β by pancreatic stellate cells. Sci Rep.

[152] Froeling FEM (2011). Retinoic acid-induced pancreatic stellate cell quiescence reduces paracrine Wnt-β-catenin signaling to slow tumor progression. Gastroenterology.

[153] Ene-Obong A (2013). Activated pancreatic stellate cells sequester CD8+ T cells to reduce their infiltration of the juxtatumoral compartment of pancreatic ductal adenocarcinoma. Gastroenterology.

[154] Kuroda H (2017). All-trans retinoic acid enhances gemcitabine cytotoxicity in human pancreatic cancer cell line AsPC-1 by up-regulating protein expression of deoxycytidine kinase. Eur J Pharm Sci.

[155] Kocher HM (2020). Phase I clinical trial repurposing all-trans retinoic acid as a stromal targeting agent for pancreatic cancer. Nat Commun.

[156] Ding N (2013). A vitamin D receptor/SMAD genomic circuit gates hepatic fibrotic response. Cell.

[157] Sherman MH (2014). Vitamin D receptor-mediated stromal reprogramming suppresses pancreatitis and enhances pancreatic cancer therapy. Cell.

[158] Wang K (2015). Expression of vitamin D receptor as a potential prognostic factor and therapeutic target in pancreatic cancer. Histopathology.

[159] Arensman MD (2015). Calcipotriol targets LRP6 to inhibit wnt signaling in pancreatic cancer. Mol Cancer Res MCR.

[160] Gorchs L (2020). The vitamin D analogue calcipotriol promotes an anti-tumorigenic phenotype of human pancreatic CAFs but reduces T cell mediated immunity. Sci Rep.

[161] Wei D (2023). Activation of vitamin D/VDR signaling reverses gemcitabine resistance of pancreatic cancer cells through inhibition of MUC1 expression. Dig Dis Sci.

[162] Schwartz GG, Eads D, Naczki C (2008). 19-nor-1 alpha,25-dihydroxyvitamin D2 (paricalcitol) inhibits the proliferation of human pancreatic cancer cells in vitro and in vivo. Cancer Biol Ther.

[163] Cheng YH (2021). Treatment of 13-cis retinoic acid and 1,25-dihydroxyvitamin D3 inhibits TNF-alpha-mediated expression of MMP-9 protein and cell invasion through the suppression of JNK pathway and microRNA 221 in human pancreatic adenocarcinoma cancer cells. PloS One.

[164] Seo Y, Baba H, Fukuda T (2000). High expression of vascular endothelial growth factor is associated with liver metastasis and a poor prognosis for patients with ductal pancreatic adenocarcinoma.

[165] Pothula SP (2016). Hepatocyte growth factor inhibition: a novel therapeutic approach in pancreatic cancer. Br J Cancer.

[166] Kemik O, Purisa S, Kemik AS (2009). Increase in the circulating level of hepatocyte growth factor in pancreatic cancer patients. Bratisl Lek Listy.

[167] Zhu GH (2011). Expression and prognostic significance of CD151, c-Met, and integrin alpha3/alpha6 in pancreatic ductal adenocarcinoma. Dig Dis Sci.

[168] Tai CJ (2017). Bevacizumab and cetuximab with conventional chemotherapy reduced pancreatic tumor weight in mouse pancreatic cancer xenografts. Clin Exp Med.

[169] Hage C (2013). The novel c-Met inhibitor cabozantinib overcomes gemcitabine resistance and stem cell signaling in pancreatic cancer. Cell Death Dis.

[170] Avan A (2013). Crizotinib inhibits metabolic inactivation of gemcitabine in c-Met-driven pancreatic carcinoma. Cancer Res.

[171] Takiguchi S, Inoue K, Matsusue K (2017). Crizotinib, a MET inhibitor, prevents peritoneal dissemination in pancreatic cancer. Int J Oncol.

[172] Brandes F (2015). Targeting cMET with INC280 impairs tumour growth and improves efficacy of gemcitabine in a pancreatic cancer model. BMC Cancer.

[173] Sahai V (2019). A phase I/II open-label multicenter single-arm study of FABLOx (metronomic 5-fluorouracil plus nab-paclitaxel, bevacizumab, leucovorin, and oxaliplatin) in patients with metastatic pancreatic cancer. J Pancreat Cancer.

[174] Kindler HL (2010). Gemcitabine plus bevacizumab compared with gemcitabine plus placebo in patients with advanced pancreatic cancer: phase III trial of the Cancer and Leukemia Group B (CALGB 80303). J Clin Oncol.

[175] Shaib WL (2023). Phase II randomised, double-blind study of mFOLFIRINOX plus ramucirumab versus mFOLFIRINOX plus placebo in advanced pancreatic cancer patients (HCRN GI14–198). Eur J Cancer.

[176] Takeuchi K, Ito F (2011). Receptor tyrosine kinases and targeted cancer therapeutics. Biol Pharm Bull.

[177] Betriu N, Andreeva A, Semino CE (2021). Erlotinib promotes ligand-induced EGFR degradation in 3D but not 2D cultures of pancreatic ductal adenocarcinoma cells. Cancers.

[178] Park R, Al-Jumayli M, Miller K (2021). Exceptional response to Erlotinib monotherapy in EGFR Exon 19-deleted, KRAS wild-type, Chemo-refractory advanced pancreatic adenocarcinoma. Cancer Treat Res Commun.

[179] Masso-Valles D (2015). Ibrutinib exerts potent antifibrotic and antitumor activities in mouse models of pancreatic adenocarcinoma. Cancer Res.

[180] Falcomatà C Selective multi-kinase inhibition sensitizes mesenchymal pancreatic cancer to immune checkpoint blockade by remodeling the tumor microenvironment. Nat Cancer.

[181] Luo L, Wang X, Liao YP (2024). Reprogramming the pancreatic cancer stroma and immune landscape by a silicasome nanocarrier delivering nintedanib, a protein tyrosine kinase inhibitor. Nano Today.

[182] Lee YE (2023). Synergistic therapeutic combination with a CAF inhibitor enhances CAR-NK-mediated cytotoxicity via reduction of CAF-released IL-6. J Immunother Cancer.

[183] Awasthi N, Schwarz MA, Schwarz RE (2011). Antitumour activity of sunitinib in combination with gemcitabine in experimental pancreatic cancer. HPB.

[184] Carter EP, Fearon AE, Grose RP (2015). Careless talk costs lives: fibroblast growth factor receptor signalling and the consequences of pathway malfunction. Trends Cell Biol.

[185] Carter EP, Coetzee AS, Tomas Bort E (2021). Dissecting FGF signalling to target cellular crosstalk in pancreatic cancer. Cells.

[186] Mancini V (2024). TRPA1 contributes to FGFR2c signaling and to its oncogenic outcomes in pancreatic ductal adenocarcinoma-derived cell lines. Cancers.

[187] Prieto-García E (2021). Tumor-stromal interactions in a co-culture model of human pancreatic adenocarcinoma cells and fibroblasts and their connection with tumor spread. Biomedicines.

[188] Rasam S, Lin Q, Shen S (2023). Highly reproducible quantitative proteomics analysis of pancreatic cancer cells reveals proteome-level effects of a novel combination drug therapy that induces cancer cell death via metabolic remodeling and activation of the extrinsic apoptosis pathway. J Proteome Res.

[189] Coetzee AS (2023). Nuclear FGFR1 promotes pancreatic stellate cell-driven invasion through up-regulation of Neuregulin. Oncogene.

[190] Moore MJ (2007). Erlotinib plus gemcitabine compared with gemcitabine alone in patients with advanced pancreatic cancer: a phase III trial of the National Cancer Institute of Canada Clinical Trials Group. J Clin Oncol.

[191] Tempero M (2021). Ibrutinib in combination with nab-paclitaxel and gemcitabine for first-line treatment of patients with metastatic pancreatic adenocarcinoma: phase III RESOLVE study. Ann Oncol.

[192] Chee CE (2013). Phase II study of dasatinib (BMS-354825) in patients with metastatic adenocarcinoma of the pancreas. The Oncologist.

[193] Deplanque G (2015). A randomized, placebo-controlled phase III trial of masitinib plus gemcitabine in the treatment of advanced pancreatic cancer. Ann Oncol.

[194] Burotto M (2023). IMscin001 Part 2: a randomised phase III, open-label, multicentre study examining the pharmacokinetics, efficacy, immunogenicity, and safety of atezolizumab subcutaneous versus intravenous administration in previously treated locally advanced or metastatic non-small-cell lung cancer and pharmacokinetics comparison with other approved indications. Ann Oncol.

[195] Tang D, Kang R (2023). Glimmers of hope for targeting oncogenic KRAS-G12D. Cancer Gene Ther.

[196] Caldas C (1994). Frequent somatic mutations and homozygous deletions of the p16 (MTS1) gene in pancreatic adenocarcinoma. Nat Genet.

